# *Drosophila* Nrf2/Keap1 Mediated Redox Signaling Supports Synaptic Function and Longevity and Impacts on Circadian Activity

**DOI:** 10.3389/fnmol.2019.00086

**Published:** 2019-04-16

**Authors:** Jereme G. Spiers, Carlo Breda, Sue Robinson, Flaviano Giorgini, Joern R. Steinert

**Affiliations:** ^1^MRC Toxicology Unit, University of Leicester, Leicester, United Kingdom; ^2^Department of Genetics and Genome Biology, University of Leicester, Leicester, United Kingdom

**Keywords:** redox signaling, Nrf2, Keap1, synaptic release, longevity, sleep, *Drosophila* neuromuscular junction

## Abstract

Many neurodegenerative conditions and age-related neuropathologies are associated with increased levels of reactive oxygen species (ROS). The cap “n” collar (CncC) family of transcription factors is one of the major cellular system that fights oxidative insults, becoming activated in response to oxidative stress. This transcription factor signaling is conserved from metazoans to human and has a major developmental and disease-associated relevance. An important mammalian member of the CncC family is nuclear factor erythroid 2-related factor 2 (Nrf2) which has been studied in numerous cellular systems and represents an important target for drug discovery in different diseases. CncC is negatively regulated by Kelch-like ECH associated protein 1 (Keap1) and this interaction provides the basis for a homeostatic control of cellular antioxidant defense. We have utilized the *Drosophila* model system to investigate the roles of CncC signaling on longevity, neuronal function and circadian rhythm. Furthermore, we assessed the effects of CncC function on larvae and adult flies following exposure to stress. Our data reveal that constitutive overexpression of CncC modifies synaptic mechanisms that positively impact on neuronal function, and suppression of CncC inhibitor, Keap1, shows beneficial phenotypes on synaptic function and longevity. Moreover, supplementation of antioxidants mimics the effects of augmenting CncC signaling. Under stress conditions, lack of CncC signaling worsens survival rates and neuronal function whilst silencing Keap1 protects against stress-induced neuronal decline. Interestingly, overexpression and RNAi-mediated downregulation of CncC have differential effects on sleep patterns possibly *via* interactions with redox-sensitive circadian cycles. Thus, our data illustrate the important regulatory potential of CncC signaling in neuronal function and synaptic release affecting multiple aspects within the nervous system.

## Introduction

One of the major cellular defense mechanisms against oxidative stress is mediated by the mammalian nuclear factor erythroid 2-related factor 2 (Nrf2) and Kelch-like ECH associated protein 1 (Keap1) signaling cascade. The Nrf2/Keap1 pathway regulates gene expression of many cytoprotective and detoxifying enzymes, thus playing a pivotal role in maintaining cellular redox homeostasis. Nrf2 belongs to the cap “n” collar (*CncC*) subfamily of basic region leucine zipper transcription factors and its regulation and importance in cellular defense mechanisms has been studied in numerous physiological and pathological conditions.

Nrf2 plays a key role in neuronal resistance to oxidative stress mediated by reactive oxygen species (ROS) and glutamate-induced excitotoxicity (He et al., 2014). Balancing oxidative stress by up-regulation of Nrf2 antioxidant defense has been demonstrated to be effective in neurodegenerative disease treatment. Aging is one of the main risk factors for neurodegenerative conditions, but it is also closely associated with a loss of Nrf2 activity. Nrf2 and expression of downstream target genes are decreased in the substantia nigra of aged rats, with Nrf2 overexpression exerting a protective response to neurodegeneration (Habas et al., 2013), including in models of amyotrophic lateral sclerosis (ALS), stroke, Alzheimer’s disease (AD) and Parkinson’s disease (PD). Indeed, Nrf2 activation has been shown to alleviate neurodegenerative symptoms in a *Drosophila* model of PD (Barone et al., 2011). Furthermore, Nrf2-mediated neuroprotection is primarily conferred by astroglia both *in vitro* and *in vivo* (Liddell, 2017) and in AD patients, Nrf2 expression is decreased in both hippocampal neurons and astrocytes (Ramsey et al., 2007) indicating a strong involvement of Nrf2 signaling in neurodegeneration and neuronal function. Previous work has shown that activation of the Nrf2/Keap1 transcriptional pathways can protect hippocampal neurons from Aβ-induced neurodegeneration in an AD mouse model (Lipton et al., 2016) and rescue neuronal deficiencies in various models for PD (Johnson and Johnson, 2015), confirming a protective role in neuronal function with potential for therapeutic treatments. However, the exact targets and mechanisms of the antioxidant activities of Nrf2/Keap1 activation in the modulation of neuronal function are not fully understood.

One important characteristic of neurodegenerative diseases and aging is dysregulation of sleep patterns which has been reported across different species ranging from fly to human (De Lazzari et al., 2018; Vanderheyden et al., 2018). Cumulative evidence demonstrates a close connection between cellular circadian rhythm and redox systems. The circadian clock is involved in the regulation of ROS levels both *in vivo* and *in vitro* (Desvergne et al., 2014; Early et al., 2018). In mammals, the circadian clock orchestrates the activities of the antioxidant defense and oxidative stress response systems by mediating Nrf2 signaling. Two proteins involved in circadian rhythm, circadian locomotor output cycles kaput (Clock) and Brain and muscle arnt-like protein-1 (Bmal1) can positively regulate Nrf2 transcription, which in turn drives rhythmic oscillations of antioxidant genes (Xu et al., 2012; Pekovic-Vaughan et al., 2014). Conversely, the cellular redox state is critically important for the regulation of Bmal1 and Clock gene transcriptional activities (Ranieri et al., 2015).

The mechanism underlying the Nrf2 protective response remains obscure and given the limited understanding of Nrf2/Keap1 signaling on neuronal function we utilized the *Drosophila* model in this study to investigate the effects on aging, synapse function, and circadian activity. *Drosophila* *Keap1* acts as a negative regulator of *CncC* (Itoh et al., 1999; Sykiotis and Bohmann, 2008; Pitoniak and Bohmann, 2015) and its silencing by RNAi leads to endogenous activation of *CncC* signaling with flies showing upregulation of the classical antioxidant response element cascade (Sykiotis and Bohmann, 2008), which increases their stress resistance. In particular, CncC activation results in enhanced transcription of the antioxidant and detoxifying enzyme glutathione S-transferase encoded by the *Drosophila* *gstD1* gene (Sawicki et al., 2003; Sykiotis and Bohmann, 2008) which acts in a neuroprotective manner.

We manipulated neuronal antioxidant response ability by either overexpressing CncC or reducing the expression of CncC and Keap1 protein by RNAi. We then investigated longevity, activity, and circadian behavior in adult flies in addition to synaptic function at the larval neuromuscular junction (NMJ). The data show that constitutive overexpression of CncC has important impacts on synaptic release and survival with silencing of the CncC inhibitor, *Keap1*, inducing beneficial effects on survival and synaptic function. Importantly, application of the antioxidant compounds dithiothreitol (DTT) and glutathione (GSH) produced similar effects to those mediated by *CncC* overexpression or *Keap1* silencing, suggesting that an antioxidant environment boosts synaptic function in a redox-specific manner.

## Materials and Methods

### Fly Husbandry

Flies were raised on standard maize media at 25°C at a 12-h LD cycle. The *elav*-GAL4[C155] driver was obtained from the Bloomington Stock Center (Indiana, US). The UAS-RNAi lines [*Keap1* (CG3962) and *CncC* (CG43286)] were purchased from the Vienna *Drosophila* Resource Centre (VDRC). The UAS-*CncC* line was kindly provided by Dirk Bohmann, University of Rochester, USA (Sykiotis and Bohmann, 2008; Pitoniak and Bohmann, 2015). The UAS/GAL4 bipartite expression system was utilized to drive pan-neuronal expression. The *elav*-GAL4 driver (female flies) and the UAS responder lines (male flies) were crossed to obtain offspring expressing the genes of interest. As a control for the RNAi strains, a line carrying an empty RNAi vector inserted in the AttP40 site was used and crossed to the *elav-*GAL4 driver (referred to as RNAi Ctrl). For *CncC* overexpressing (OE) lines, experimental lines were compared to control obtained by crossing the GAL4 (*elav* Ctrl) and UAS lines to *w*^1118^. The homozygote *w*^1118^ line was used as a control in the pharmacology experiments.

### Electrophysiology

TEVC recordings were performed as described previously (Robinson et al., 2018). Sharp-electrode recordings were made from ventral longitudinal m6 in abdominal segments 2 and 3 of third instar larvae using pClamp 10.5, an Axoclamp 900A amplifier and Digidata 1440A (Molecular Devices, US) in hemolymph-like solution 3 (HL-3; Stewart et al., 1994). Recording electrodes (20–50 MΩ) were filled with 3 M KCl. Miniature excitatory junctional currents (mEJCs) were recorded in the presence of 0.5 μM tetrodotoxin (Tocris, UK). All synaptic responses were recorded from muscles with input resistances ≥4 MΩ, holding currents <4 nA at −60 mV and resting potentials more negative than −60 mV at 25°C, as differences in recording temperature cause changes in glutamate receptor kinetics and amplitudes (Postlethwaite et al., 2007). Holding potentials were −60 mV. The extracellular HL-3 contained (in mM): 70 NaCl, 5 KCl, 20 MgCl_2_, 10 NaHCO_3_, 115 sucrose, 5 trehalose, 5 HEPES, and 1.5 CaCl_2_. Average single evoked EJC (eEJC) amplitudes (stimulus: 0.1 ms, 1–5 V) were based on the mean peak eEJC amplitude in response to 10 presynaptic stimuli (recorded at 0.2 Hz). Nerve stimulation was performed with an isolated stimulator (DS2A, Digitimer). All data were digitized at 10 kHz and for miniature recordings, 200-s recordings were analyzed to obtain mean mEJC amplitudes. The quantal content (QC) was estimated for each recording by calculating the ratio of eEJC amplitude/average mEJC amplitude, followed by averaging recordings across all NMJs for a given genotype. mEJC and eEJC recordings were off-line low-pass filtered at 500 Hz and 1 kHz, respectively. Materials were purchased from Sigma-Aldrich (UK).

### Cumulative Postsynaptic Current Analysis

The apparent size of the RRP was probed by the method of cumulative eEJC amplitudes (Schneggenburger et al., 1999). Muscles were clamped to −60 mV and eEJC amplitudes during a stimulus train [50 Hz, 500 ms (of a 1-s train)] were calculated as the difference between peak and baseline before stimulus onset of a given eEJC. Receptor desensitization was not blocked as it did not affect eEJC amplitudes, because a comparison of the decay of the first and the last eEJC within a train did not reveal any significant difference in decay kinetics. The number of release-ready vesicles (*N*) was obtained by back extrapolating a line fit to the linear phase of the 500-ms cumulative eEJC plot (the last 200 ms of the train) to time zero by dividing the cumulative eEJC amplitude at time zero by the mean mEJC amplitude recorded in the same cell. To calculate the QC in the train, we used mean mEJC amplitudes measured before the train.

### Heat Shock Protocol

For heat shock survival experiments, methods were adapted from Ishida et al. (2012). Briefly, male adult flies (aged 3–5 days) were transferred to vials containing a moistened filter pad to prevent dehydration. Vials were placed in a 37°C water bath and live flies were counted every 30 min. In larval experiments, heat shock was induced using previously described methods (Robinson et al., 2017). Briefly, age-matched third instar larvae were incubated at 37°C for 1 h and used for electrophysiology 24 h later. These experiments were repeated a minimum of three times for each genotype.

### Circadian

Circadian activity and sleep analysis were performed as described previously (Ishida et al., 2012). Briefly, adult male flies (aged 3–5 days) were individually transferred into glass tubes containing food. Single tubes were then loaded into *Drosophila* Activity Monitor system (Trikinetics). Following a 2 days period of entrainment in incubators kept on a 12:12 light/dark regime at 25°C, locomotor activity was recorded for the consecutive 5 days. Sleep behavior was analyzed by using pySolo software (Gilestro and Cirelli, 2009).

### Survival

Groups of 10 newly emerged adult male flies were transferred to new vials containing food and deaths were scored daily. Flies were transferred to new food three times per week and otherwise left undisturbed. Cumulative survival curves are presented and compared using the Log-rank (Mantel-Cox) test.

### Geotaxis

Rapid iterative negative geotaxis behavior was performed using methods outlined previously (Rhodenizer et al., 2008; Nichols et al., 2012). Briefly, age-matched adult male flies were collected and groups of 10 were transferred to a clear empty vial without anesthesia at weekly intervals. Tubes were transferred to a quiet room and flies acclimated for 15 min. Tubes were tapped three times on a bench and images were taken after 3 s using a digital camera. A minimum of five trials were conducted per session with an inter-trial interval of 1 min. Average height climbed per vial was calculated from images using Image J software. These experiments were repeated a minimum of three times per genotype.

### Crawling Activity

Age-matched third instar male larvae (~100–120 h) were selected, washed and placed onto a moist, food-free surface at a constant temperature of 20°C. Crawling activities were imaged over 10 min using AnyMaze software v4.98 (Stoelting Co., Wood Dale, IL, USA) and data were analyzed off-line as reported previously (Robinson et al., 2014).

### Statistics

Statistical analysis was performed with Prism 7 (Graphpad Software Inc., San Diego, CA, USA). Statistical tests were carried out using a one-way ANOVA test when applicable with a posteriori test (with Tukey’s multiple comparisons) or unpaired Student’s *t*-test [for comparisons between *elav* × *CncC* and *elav* × *w*^1118^ (*elav* Ctrl)]. Data in figures are expressed as mean ± SEM where *n* is the number NMJs, flies or larvae as indicated, and significance is shown as *p < 0.05, **p < 0.01, ***p < 0.001, and ****p < 0.0001.

## Results

**Figure 1:**
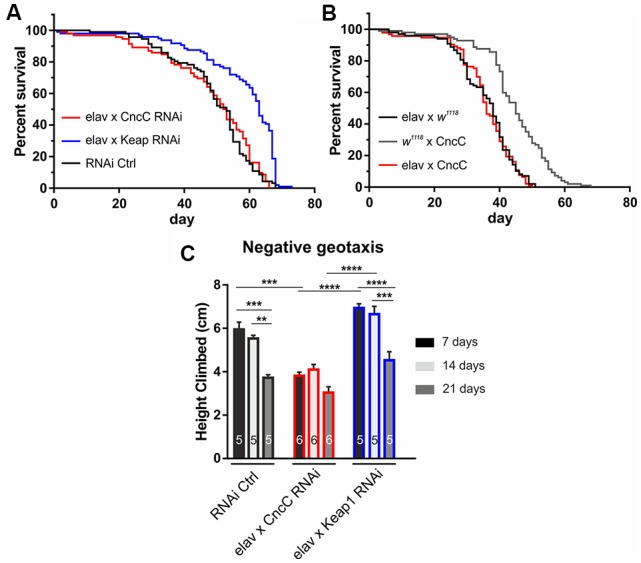
Suppression of Kelch-like ECH associated protein 1 (*Keap1*) signaling extends life span and promotes negative geotaxis activity. **(A)** RNAi silencing of *Keap1* prolongs life span, whereas *CncC* downregulation does not affect longevity (*n* = 60–98 flies, *p* < 0.0001). **(B)** Overexpression of *CncC* does not affect life span (*n* = 99–72 flies, *p* > 0.05). Data were compared using the Log-rank (Mantel-Cox) test. **(C)** Negative geotaxis performance declines with age in control flies (RNAi Ctrl at 21 days, dark gray), however, *CncC* RNAi expression induces a strong reduction in climbing activity at 7 days (black) with no further effects at older ages. RNAi silencing of *Keap1* augments activity decline relative to *CncC* silencing at seven (black) and 14 (light gray) days (*n*—number of flies indicated within bars). Data denote mean ± SEM for all data comparisons in **(C)**. One-way ANOVA with *post hoc* Tukey-Kramer was used for comparisons with **p < 0.01, ***p < 0.001, ****p < 0.0001.

We used this well-characterized expression system and first assessed the effects of down-regulating either CncC or Keap1 protein expression on *Drosophila* life span as it has long been postulated that oxidative stress contributes to age-related neuronal dysfunction known as the free radical theory of aging. Under unchallenged conditions, the suppression of Keap1 expression (*Keap1-*RNAi) causes an increase in longevity, whereas CncC silencing (*CncC-*RNAi) does not show any effects [median life span (in days): RNAi Ctrl (*elav* GAL4, UAS RNAi Control): 53, *CncC*-RNAi: 52.5, *Keap1*-RNAi: 63, *p* < 0.0001, Log-rank (Mantel-Cox) test, Figure 1A] implicating that the lack of CncC signaling might be compensated by other defense mechanisms, such as the thioredoxin system (Radyuk et al., 2003). Moreover, pan-neuronal overexpression (OE) of *CncC* did not cause any significant improvements in life span of unstressed flies relative to their controls [median life span (in days): *elav* × *CncC*: 36, *elav* × *w*^1118^: 38, *w*^1118^ × *CncC*: 45, *p* > 0.05, Log-rank (Mantel-Cox) test, Figure 1B] which has previously been reported (Pitoniak and Bohmann, 2015) and could be the result of endogenous suppression of CncC nuclear translocation and transcriptional activity similar to that reported for Nrf1 signaling (Wang and Chan, 2006). In addition to longevity studies, fly aging can also be evaluated by geotaxis-driven activity assays, leading us to assess the activity of adult *Drosophila* individuals with *CncC* and *Keap1* silencing at 7, 14 and 21 days of age. As CncC OE did not alter longevity we did not further use this genotype in this assay. *CncC* silencing reduced negative geotaxis-mediated activity (7 days: RNAi Ctrl: 5.9 ± 0.3 cm; *CncC*-RNAi: 3.8 ± 0.2 cm, *Keap1*-RNAi: 6.9 ± 0.2 cm, Figure 1C), whereas *Keap1-*RNAi individuals displayed enhanced performance relative to *CncC* silencing (14 days: RNAi Ctrl: 5.5 ± 0.2 cm; *CncC*-RNAi: 4.1 ± 0.2 cm, *Keap1*-RNAi: 6.6 ± 0.3 cm, 21 days: RNAi Ctrl: 3.7 ± 0.1 cm; *CncC*-RNAi: 3.0 ± 0.2 cm, *Keap1*-RNAi: 4.5 ± 0.4 cm, ANOVA).

To further characterize the effects on survival and geotaxis-driven activity and their connection to neuronal health, we assessed neuronal function in more detail in electrophysiological experiments of the larval NMJ. This well-studied synapse allows direct assessment of neuronal health and synaptic function by recording single action potential-eEJC, spontaneous release events (mEJC), and total vesicular pool size release. Oxidative stress and aging have been related to compromised neuronal function and diminished synaptic release. However, to our knowledge, the direct effects of CncC/Keap1 signaling on synaptic release have not yet been documented (Besson et al., 2000; Fremeau et al., 2004; Escartin et al., 2011; Wu and Cooper, 2012; Cirillo et al., 2015; Ivannikov and Van Remmen, 2015).

**Figure 2:**
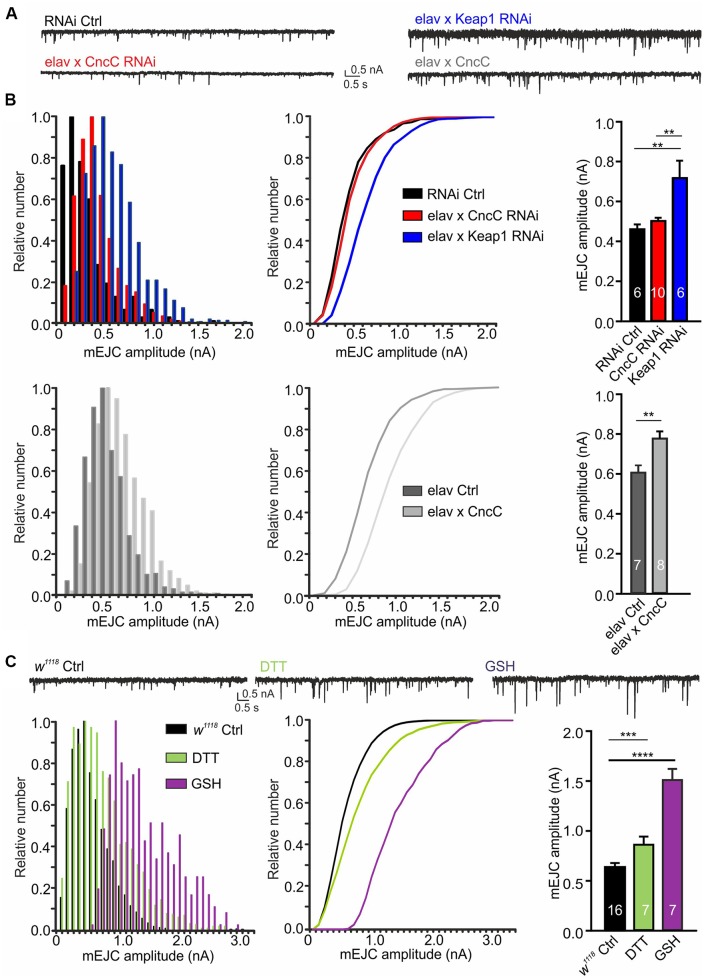
Upregulation of antioxidant signaling enhances spontaneous quantal release at the neuromuscular junction (NMJ) synapse. **(A)** Example recordings of spontaneous activity at larval NMJs. **(B)** Overexpression of *CncC* and silencing of *Keap1* enhances the quantal size, illustrated in amplitude frequency plots for miniature excitatory junctional current (mEJC) amplitudes (left), cumulative amplitude frequency plots (middle) and mean bar graphs (right). **(C)** Exposure to antioxidants dithiothreitol (DTT) and glutathione (GSH) induced a strong increase in quantal size at NMJs of *w*^1118^ larvae as illustrated in frequency plots for mEJC amplitudes (left), cumulative amplitude frequency plots (middle) and mean bar graphs (right). One-way ANOVA with *post hoc* Tukey-Kramer was used for comparisons with **p < 0.01, ***p < 0.001, ****p < 0.0001 [*n*—number of NMJs (from at least three larvae) indicated within bars].

Interestingly, our studies showed that enhancing antioxidant potential *via* upregulation of *CncC* signaling by either *Keap1* silencing or *CncC* overexpression (OE), potently increased mEJC amplitudes [RNAi Ctrl: 0.46 ± 0.02 nA, *CncC*-RNAi: 0.50 ± 0.02 nA, *Keap1-*RNAi: 0.72 ± 0.09 nA (ANOVA); *CncC* OE: 0.78 ± 0.04 nA, *elav* Ctrl: 0.61 ± 0.03 nA (Student’s *t*-test), Figures 2A,B]. Confirmatory studies further showed treatment with the antioxidants DTT (1 mM DTT; ROS scavenger) or reduced GSH (150 μM GSH) for 45 min significantly enhanced mEJC amplitudes [*w*^1118^: 0.62 ± 0.02 nA, DTT: 0.86 ± 0.08 nA, GSH: 1.51 ± 0.11 nA, ANOVA, Figure 2C], further corroborating the effects seen following genetic manipulation of antioxidant signaling pathways and indicating that enhanced antioxidant levels at the synapse promote larger quantal size.

**Figure 3:**
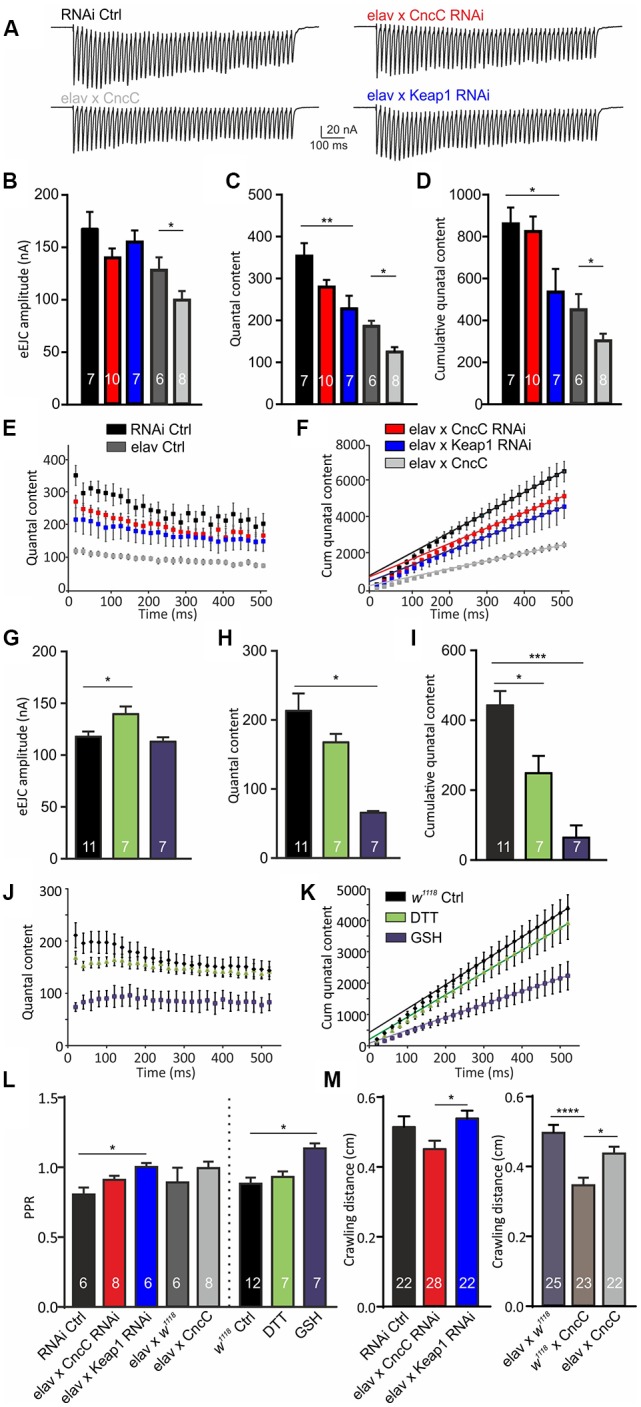
Increased antioxidant environment allows for reduction of stimulated vesicular release while maintaining synaptic transmission. **(A)** Recordings of evoked synaptic currents at the larval NMJ. Graphs show means for evoked EJC (eEJC) amplitudes **(B)**, quantal content (QC; **C**) and cumulative QC **(D)**, QC and cumulative QC graphs of 50 Hz trains for genotypes indicated **(E,F)**. eEJC amplitudes **(G)**, QC **(H)** and cumulative QC **(I)** for treatments indicated. **(J,K)** Graphs of 50 Hz trains from *w*^1118^ larvae and *w*^1118^ larvae treated with GSH and DTT. **(L)** Paired-pulse ratios (PPR) were analyzed at 20 ms inter-spike intervals at NMJs from indicated genotypes and/or treatments [ANOVA used for comparison of RNAi expressing strains, Student’s *t*-test was used to compare elav × CncC with elav Ctrl, *n*—number of NMJs (from at least three larvae) indicated within bars]. **(M)** Mean crawling distances of different genotypes over a 10 min imaging period (*n*—number of larvae indicated within bars). One-way ANOVA with *post hoc* Tukey-Kramer was used for comparisons with *p < 0.05, **p < 0.01, ***p < 0.001, ****p < 0.0001.

However, when analyzing evoked EJC (eEJC) amplitudes, we did not detect any increase in amplitudes following genetic induction of antioxidant signaling but unexpectedly a decrease in eEJC amplitudes following CncC OE [RNAi Ctrl: 165 ± 17 nA, *CncC*-RNAi: 140 ± 9 nA, *Keap1*-RNAi: 151 ± 12 nA (ANOVA); *CncC* OE: 99 ± 8 nA, *elav* Ctrl: 127 ± 12 nA (Student’s *t*-test), Figures 3A,B]. Although this was somewhat surprising, however, given the fact that this synapse is under high homeostatic regulation (Müller et al., 2012; Frank, 2014; Li et al., 2018), it is likely that elevated quantal size (mEJC amplitude) leads to a reduced QC of the evoked response thereby leaving the evoked amplitudes unchanged or even decreased. This homeostatic feedback pathway regulates release within relatively short time frames (tens of minutes) and provides a rapid physiological regulatory mechanism. In fact, when assessing the QC we found that both *CncC* OE and knock-down (KD) of *Keap1* induced a decrease in QC [RNAi Ctrl: 356 ± 33, *CncC*-RNAi: 278 ± 16, *Keap1*-RNAi: 225 ± 32 (ANOVA); *CncC* OE: 122 ± 8, *elav* Ctrl: 184 ± 13 (Student’s *t*-test), Figures 3C,E]. This reduction in release induced a strong effect on the total vesicular release capacity (vesicle pool size) of the NMJ, with the calculated number of releasable quanta per NMJ (as calculated by the cumulative postsynaptic current analysis of a 50 Hz train yielding the cumulative QC) being drastically reduced following *CncC* OE or *Keap1-*RNAi expression [RNAi Ctrl: 837 ± 97, *CncC*-RNAi: 820 ± 72, *Keap1*-RNAi: 531 ± 110 (ANOVA); *CncC* OE: 298 ± 34, *elav* Ctrl: 459 ± 64 (Student’s *t*-test), Figures 3D,F]. To exclude the possibility that genetic manipulation caused developmental changes which could interfere with our data interpretation, we again assessed the effects of pharmacological manipulation of redox signaling on release parameters of the synapse. Quantification of NMJ evoked responses and QC revealed similar changes using antioxidant supplementation as following genetic manipulations. DTT application for 45 min induced mild effects and GSH application for the same length led to strong effects on the three parameters [eEJC amplitude: *w*^1118^: 118 ± 5 nA, DTT: 140 ± 7 nA, GSH: 113 ± 4 nA, QC: *w*^1118^: 213 ± 25, DTT: 167 ± 12, GSH: 65 ± 3, cumulative QC: *w*^1118^: 441 ± 40, DTT: 247 ± 49, GSH: 63 ± 35, ANOVA, Figures 3G–K] compared to controls (*w*^1118^), similar to the changes observed following genetically-induced increase in antioxidant potential suggesting an acute mechanism mediated by a reduction in basal oxidative stress levels. Importantly, *CncC* KD was without effects indicating that under these unchallenged conditions the deficiency in potential antioxidant capacity did not alter basal synaptic transmission. One important regulatory mechanism with the ability to modulate synaptic release is the control of synaptic release probabilities. The initial release probability can be adjusted in response to various mechanisms. We assessed potential effects on the initial vesicular release probability (p_vr_) by measuring paired-pulse ratios (PPR) at a 20 ms inter-spike interval. Previously we found that the nitrergic regulation of synaptic release at the NMJ is mediated *via* a reduction of p_vr_ (Robinson et al., 2014) as manifested in an increased PPR. Paired-pulse experiments at larval NMJs revealed a significant increase in PPR following genetic alterations of *Keap1* expression suggesting that p_vr_ is reduced at lower oxidative stress levels [RNAi Ctrl: 0.81 ± 0.05, *CncC*-RNAi: 0.92 ± 0.02, *Keap1*-RNAi: 0.99 ± 0.02 (ANOVA); *CncC* OE: 1.00 ± 0.05, *elav* Ctrl: 0.88 ± 0.11 (Student’s *t*-test), Figure 3L]. However, following pharmacological modulation of redox signaling we detected an increase in PPR only after GSH application [*w*^1118^: 0.88 ± 0.04, DTT: 0.92 ± 0.04, GSH: 1.12 ± 0.05, ANOVA, Figure 3L]. Importantly, changes in frequency (*f*) of spontaneous release indicates direct effects on vesicle fusion mediated by the soluble *N*-ethyl-maleimide-sensitive fusion protein Attachment Protein Receptor (SNARE) and SNARE binding proteins. To investigate the modulation of these mechanisms, we measured mEJC frequencies. The results did not show any differences in *f* between larvae [RNAi Ctrl: 2.6 ± 0.4 s^−1^, *CncC*-RNAi: 3.2 ± 0.3 s^−1^, *Keap1*-RNAi: 3.4 ± 0.5 s^−1^ (ANOVA); *CncC* OE: 1.4 ± 0.2 s^−1^, *elav* Ctrl: 3.14 ± 0.80 (Student’s *t*-test); *w*^1118^: 2.0 ± 0.2 s^−1^, GSH: 1.2 ± 0.2 s^−1^, DTT: 3.4 ± 0.3 s^−1^ (ANOVA) *p* > 0.05] suggesting that vesicle fusion mechanisms *per se* are not modulated by changes in the redox level following genetic or pharmacological manipulations.

Together, our data show that regulation of the redox environment can alter synaptic function with spontaneous release events being positively modulated in a low oxidative stress environment. We next wondered if the observed regulation of synaptic release could translate into changes of larval activity. To test larval activity, we assessed crawling distances of the different genotypes over a period of 10 min (Robinson et al., 2014). Since motoneuronal transmission during crawling activity is predominately related to single motoneuronal action potential–induced synaptic release, which corresponds to a single eEJC event, we would not expect major effects on larval activity. Indeed, neither activation nor suppression of CncC signaling affected larval crawling distances relative to controls [RNAi Ctrl: 0.5 ± 0.03 cm, *CncC*-RNAi: 0.4 ± 0.02 cm, *Keap1*-RNAi: 0.5 ± 0.02 cm, *CncC* OE: 0.4 ± 0.02 cm, *elav* × *w*^1118^: 0.5 ± 0.02 cm, *w*^1118^ × CncC: 0.3 ± 0.02 cm, ANOVA, Figure 3M] but the data showed subtle differences between *CncC-*RNAi and *Keap1-*RNAi expressing larvae.

**Figure 4:**
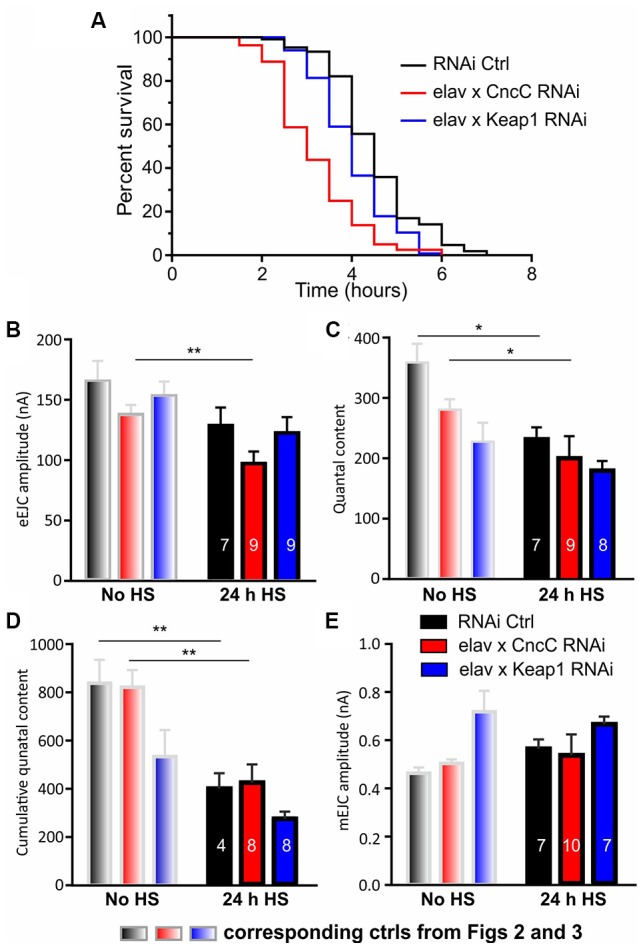
Silencing of *Keap1* protects against stress-induced synaptic decline. Life spans were analyzed for indicated lines and survivorship was plotted over time. **(A)** Survival curves represent an average of three life-span trials (*n* = 80–106 flies). Data were compared using the Log-rank (Mantel-Cox) test, *p* < 0.0001). Synaptic function was analyzed showing eEJC amplitudes **(B)** QC **(C)**, cumulative QC **(D)** and mEJC amplitudes **(E)** under control [no heat shock (no HS), gray] and heat shock challenged (24 h HS) conditions. Note that the bars in gray are repeats from Figures 2; 3 and comparisons were made for each genotype before and after HS using the unpaired Student’s *t*-test with *p < 0.05, **p < 0.01 [*n*—number of NMJs (from at least three larvae) indicated within bars].

Increased oxidative stress levels are caused by altered activities of thiol redox circuits that can result in impaired cell signaling and dysfunctional redox-control (Finkel, 2011). It is linked to several pathological processes including dysfunction of proteostasis and the accumulation of misfolded proteins in the lumen of the endoplasmic reticulum (ER), resulting in ER stress (Braakman and Hebert, 2013). As heat shock is involved in triggering ER stress and ROS signaling, we next wanted to test whether altered expression of CncC and Keap1 would affect *Drosophila* longevity and synapse function following heat shock challenge. We found that under continual heat stress-challenged conditions, KD of *CncC* drastically reduced longevity [median life span (in hours): RNAi Ctrl: 4.5, *CncC*-RNAi: 3, *p* < 0.0001, Log-rank (Mantel-Cox) test] which can be explained by lack of neuroprotective Nrf2 signaling (Ahmed et al., 2017), while expectedly, *Keap1* silencing protected and led to increased survival compared to *CncC* KD [median life span (in hours): *Keap1*-RNAi: 4, *p* < 0.0001, Log-rank (Mantel-Cox) test, Figure 4A]. The neuroprotection mediated by CncC (Nrf2) activation was further assessed in heat shock challenged larvae in which we characterized synaptic responses at the NMJ. The most striking changes at the level of synapse physiology occurred in *CncC* KD larvae 24 h after a single 1 h of heat shock challenge in which eEJC amplitudes declined drastically. This physiological response was not due to changes in quantal size but rather due to a reduced quantal content [eEJC amplitude: RNAi Ctrl: 112 ± 9 nA, *CncC*-RNAi: 74 ± 4 nA, *Keap1*-RNAi: 103 ± 9 nA, QC: RNAi Ctrl: 200 ± 15, *CncC*-RNAi: 186 ± 33, *Keap1*-RNAi: 142 ± 15, mEJC amplitude: RNAi Ctrl: 0.56 ± 0.04 nA, *CncC*-RNAi: 0.54 ± 0.09 nA, *Keap1*-RNAi: 0.66 ± 0.03 nA, Figures 4B,C,E]. Importantly, control larvae also showed a reduction in QC following challenge with heat shock, with *Keap1* KD, however, preventing further neuronal deterioration upon this challenge. These changes were consolidated by measuring the vesicle pool size as cumulative release following synaptic stimulation in trains at 50 Hz [cumulative QC: RNAi Ctrl: 398 ± 64, *CncC*-RNAi: 423 ± 75, *Keap1*-RNAi: 272 ± 30, Figure 4D]. The data suggest that following heat shock stimulation, the lack of *CncC* produces strong phenotypes with regards to longevity and synapse function which were partially observed in controls but abolished following *Keap1* KD.

Many neurological conditions including AD and PD exhibit perturbations of the circadian system (sometimes prior to any motor symptoms or clinical manifestation of symptoms), and underlying pathways have been studied in various animal models (Videnovic et al., 2014). Light and temperature are the two most reliable environmental timing cues, referred to as Zeitgeber (ZT), for the resetting of circadian clocks (Pittendrigh, 1960; Buhr et al., 2010; Musiek et al., 2013; Tamaru et al., 2013). Notably, mRNA expression levels for Keap1a/b and Nrf2 vary significantly within 12 h (i.e., between ZT0 and ZT12) implicating their involvement in circadian redox regulation (Zheng et al., 2017). This prompted us to evaluate how changes in ROS levels would impact the circadian behavior of flies with reduced or augmented cellular antioxidant capacity in a 24-h light-dark (LD) cycle. To determine how the circadian system is affected by modulating ROS levels *via* CncC/Keap1 signaling, we measured activity and sleep pattern as an index of circadian behavior in flies with reduced or augmented cellular antioxidant capacity.

**Figure 5:**
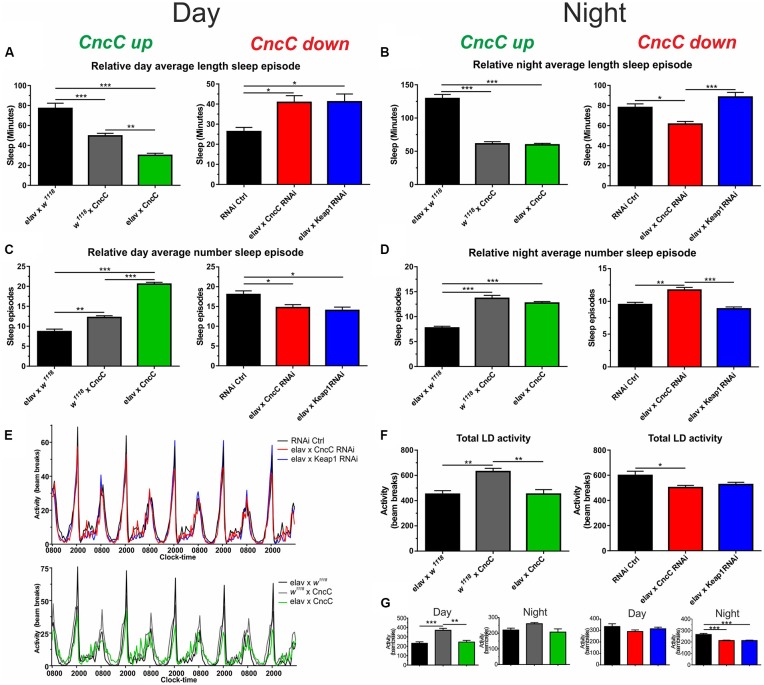
The CncC/Keap1 signaling impacts on sleep and activity patterns. **(A,B)** Sleep duration (in min) was reduced following *CncC* OE and enhanced in *CncC* knock-down (KD) flies in the day time, during the night, *CncC* KD reduced sleep length. The overexpression of *CncC* caused fragmented sleep patterns in the day resulting in more but shorter sleep episodes in the night time **(B)**, whereas *CncC* KD reduces sleep length with more episodes in the night; **(C,D)** *Keap1* silencing reverses the *CncC* KD effect in the night but not in the day. **(D)** During the night, both *CncC* OE and KD induce similar changes resulting in more but shorter sleep episodes. **(E–G)** Total and night/day activities of genotypes. One-way ANOVA with *post hoc* Tukey-Kramer was used for comparisons with *p < 0.05, **p < 0.01, ***p < 0.001 [*n*—numbers: elav × W, 3M (RNAi Ctrl: 38), elav × *CncC* RNAi: 21, elav × *Keap1* RNAi: 44, elav × *W*^1118^: 32, *W*^1118^ × *CncC*: 27, elav × *CncC*: 25].

Quantification of sleep episodes in the day and night should give the best overall picture of sleep behavior. We quantified the relative length and number of sleep episodes and found that in the light phase *CncC* OE reduces the length of sleep but enhances the number of sleep episodes, an effect that was reversed with silencing of *CncC* (Figures 5A,C). Unexpectedly, following *Keap1* KD, sleep behavior was similar to *CncC* KD flies in this phase. In the scotophase (dark), the overexpression of *CncC* did not cause any change in sleep parameters which were similar to the controls (Figures 5B,D). Interestingly, the behavior of *CncC* KD flies in the dark phase was different from the one observed in the photophase (light), showing a decreased length of sleep episodes and an increase in the number of these episodes suggesting that driving redox levels in one direction has opposite effects on sleep depending on the time. Conversely, sleep behavior of *Keap1* KD flies was more similar to that observed in the photophase (light), showing only a significant increase in the sleep length episodes (Figures 5C,D). Finally, we measured the total activity of adult flies and found that total activity was only reduced in *CncC* KD flies in comparison to controls and *Keap1* KD (Figures 5E,F). Figure 5G separates the total activity profiles for the studied genotypes into day and night phases showing that *CncC* but also *Keap1* KD reduced activity in the dark phase only. These data imply complex interactions between ROS signaling which causes differential effects over the circadian cycle.

In summary, our data provide new evidence of how regulation of the redox homeostasis *via* modulation of CncC/Keap1 signaling can modulate aging, synapse function and sleep behavior. Specifically, the suppression of *Keap1* expression induced beneficial effects on survival and synapse function. Equally, overexpression of *CncC* and pharmacologically enhancing antioxidant signaling resulted in similar phenotypes, with increases in quantal release being a major result of lowered oxidative stress signaling. The changes in synaptic function can further impact redox-sensitive aspects of sleep behavior which is implicated in disease-associated defects of circadian rhythm in neurodegeneration.

## Discussion

*Drosophila* has been instrumental in studying synapse function but also modeling various neurodegenerative diseases, including polyglutamine expansion diseases, α-synuclein-linked PD, and other prionopathies and tauopathies (see review McGurk et al., 2015). We and others have previously shown that expression of huntingtin with polyglutamine expansions, mutant α-synuclein, Aβ_40/42_ toxicity and prion-mediated pathology suppresses glutamatergic function at the NMJ and causes neurodegenerative phenotypes (Outeiro et al., 2007; Romero et al., 2008; Chakraborty et al., 2011; Steinert et al., 2012; Breda et al., 2015; Vicente Miranda et al., 2016; Fernandez-Funez et al., 2017; Martin-Peña et al., 2017). Furthermore, studies in fly have found that dysfunctional superoxide dismutase 1 (SOD1) activity associated with enhanced oxidative stress can impact upon synapse function. In particular, SOD1 mutant flies exhibit signs of neurodegeneration, locomotor deficits, and shortened life span (Sahin et al., 2017). The *Drosophila* NMJ specifically offers a unique model synapse to study regulatory mechanisms of vesicular release. However, the direct effects of the redox signaling mediated by the Nrf2/Keap1 cascade have not yet been fully assessed at the level of synapse function and whole animal behavior. Our data present new evidence of how CncC and Keap1 signaling modulates *Drosophila* longevity, synapse function, and larval and adult fly activities, including effects on circadian sleep patterns. Furthermore, we determined the protective effects of CncC/Keap1 activity under stress-challenged conditions.

Many neurodegenerative diseases are characterized by a slowly progressive loss of neurons. The etiology of these diseases has still not yet been fully elucidated, although elevated levels of oxidative stress have been suggested as one of the potential common factors. One disease, in particular, is associated with defects in the antioxidant system, with mutations in SOD1 being a strong contributor to ALS. *Drosophila* has been utilized to characterize the effects of ALS-relevant mutant proteins (Milton et al., 2011; Coyne et al., 2017; Kim et al., 2018) and induction of homeostatic neuronal plasticity can reverse ALS-induced degeneration at the NMJ (Kim et al., 2018).

Data indicate that the abnormal excitotoxic glutamate release in the spinal cord of pre-symptomatic ALS mice is mainly based on the increased size of the readily releasable pool of vesicles and release facilitation, supported by plastic changes of specific presynaptic mechanisms (Bonifacino et al., 2016). As ALS is characterized by enhanced cytotoxic oxidative stress, one could speculate that these conditions favor non-physiological release of neurotransmitter. In light of our data which show a strong reduction in the number of evoked quantal release events with an increased quantal size following augmented antioxidant signaling (GSH, *Keap1-*RNAi, *CncC* OE), we suggest that low oxidative stress balances release with a reduction in the number of energy-demanding vesicular release events and simultaneously increased quantal size to sustain physiological action potential-evoked synaptic responses. If one considers that a single vesicular release event requires around 60,000 ATP molecules at a glutamatergic synapse in the mammalian central nervous system (CNS), including neurotransmitter refilling, SNARE protein assemble/dissemble, ion pump activities (Attwell and Laughlin, 2001; Harris et al., 2012) and vesicular release represents the highest energy burden to the presynapse (Rangaraju et al., 2014), it is conceivable to suggest that low oxidative stress levels lead to advantageous low QC/high quantal size release parameters.

A great variety of factors controls the level of neurotransmitter within the vesicle and changes in vesicle filling thus have great potential to influence synaptic transmission with vesicular glutamate filling determining quantal size (Karunanithi et al., 2002; Wu et al., 2007; Huang and Trussell, 2014; Choudhury et al., 2016). Increases in *Drosophila* larval quantal size at the NMJ have been reported following >30 min of enhanced activity (Steinert et al., 2006). Conversely, high-frequency stimulation of the NMJ results in a strong decrease in quantal size (Doherty et al., 1984; Naves and Van der Kloot, 2001) illustrating the ability of this particular synapse which is under strong homeostatic control (Newman et al., 2017), but also others (see review Edwards, 2007), to modulate quantal release. It has been shown that for instance, overexpression of vesicular glutamate transporter (vGLUT) leads to larger quantal sizes and resulting reduced QC (Daniels et al., 2004). Further mechanisms include uptake of transmitter *via* the Na^+^-dependent excitatory amino acid transporters (EAATs; Wang and Floor, 1998; Takayasu et al., 2005; Rose et al., 2018), glutamate recycling which includes the glutamine-glutamate cycle, and transmitter transport into the vesicle which involves the exchange of lumenal H^+^ for cytoplasmic transmitter and hence depends on a H^+^ electrochemical gradient, that is produced by the vesicular (H^+^)-vATPase (Cotter et al., 2015). In fact, it has been shown that DTT or reduced GSH reverse H_2_O_2_-induced inhibition of the vATPase, suggesting that the mechanism of its inhibition by H_2_O_2_ involves oxidation of a reactive cysteine sulfhydryl group in the ATP binding site. Inhibition of vATPase activity would decrease the amount of transmitter stored in synaptic vesicles and thus reduce the quantal size during episodes of oxidative stress (Wang and Floor, 1998).

EAAT transporters contain cysteine-associated sulfhydryl groups sensitive to free radical species. The actions of free radicals result in the formation of cysteine bridges, thereby inhibiting glutamate transport into the cells (Trotti et al., 1998), as demonstrated for superoxide anion, hydrogen peroxide, NO and peroxynitrite (Pogun et al., 1994; Volterra et al., 1994) or into vesicles by reducing vGLUT activities (Wang et al., 2017). Conversely, overexpression of SOD1 protected glutamate transporters from inhibition (Chen et al., 2000). Studies in SOD1 knock-out mice NMJs found reduced quantal size (equal to mEJC) following enhanced oxidative stress leading to weakening of the muscle (Ivannikov and Van Remmen, 2015). Conversely, by reducing oxidative stress in a mouse model of peripheral nerve injury, the authors found increases in expression of vGLUT (Cirillo et al., 2015), the predominate vGLUT responsible for vesicular glutamate filling at the *Drosophila* NMJ (Wu and Cooper, 2012) and mammalian CNS synapses (Fremeau et al., 2004). Previous studies confirmed that activation of the Nrf2 pathway led to upregulation of the neuronal EAAT3 in mice (Escartin et al., 2011). This transporter has a homologue in *Drosophila* (Besson et al., 2000) and its upregulation enhances antioxidant activity *via* increases in glutathione production in addition to allowing sustained presynaptic glutamate levels available for release.

Conceivably, modulation of redox levels might impact on any of the above mechanisms and regulate transmitter release in a negative or positive direction and our data describe how reducing redox stress, either genetically or pharmacologically, leads to enhanced quantal size. However, this increase in quantal size resulted in a reduction of QC, likely due to homeostatic feedback regulation of this highly plastic synapse (Frank, 2014; Li et al., 2018) and future studies will have to evaluate the specific mechanisms by which redox signaling can alter vesicular transmitter release.

The observed changes of neuronal function will have wide implications on animal behavior and together with reported redox-mediated regulation of circadian function, determined by Nrf2/CncC-Keap1 signaling, our data show further evidence how this cascade can influence circadian rhythm. Neurodegeneration causes abnormalities in sleep patterns, partially due to neuronal loss but likely also due to specific dysregulation of circadian circuits. We demonstrated that increases in CncC expression or its silencing results in an opposite alteration in sleep episode length in the day. However, both conditions resulted in similar effects on sleep length during the night time. Total 24-h activity following CncC overexpression was not altered, whereas CncC KD reduced overall activity. These observations are further complicated with the expression of *Nrf2* falling under the transcriptional regulation of the Clock/Bmal1-complex (Xu et al., 2012; Pekovic-Vaughan et al., 2014). Clock/Bmal1-dependent *Nrf2* regulation gives rise to diurnal patterns in Nrf2 signaling, which underlies the rhythmic expression of antioxidant and metabolic enzymes reported in different cellular systems (Xu et al., 2012; Wang et al., 2018; Ishii et al., 2019). In this context it has been reported that Nrf2 gain- and loss-of-function affect circadian gene expression and rhythmicity in mammalian cellular systems, indicating the coupling of Nrf2 and Clock and the role of Nrf2 to integrate cellular redox status into timekeeping (Wible et al., 2018).

Collectively, this study and previous work illustrate a key mechanistic link between circadian oscillations in redox balance and Clock gene expression rhythms. However, key questions on the complex bidirectional regulation of ROS by circadian activity and vice versa remain to be answered in future studies.

## Author Contributions

JRS and JGS designed the study and wrote the manuscript. JRS, JGS, SR and CB conducted and analyzed the data. All authors contributed to, read and approved the final manuscript.

## Conflict of Interest Statement

The authors declare that the research was conducted in the absence of any commercial or financial relationships that could be construed as a potential conflict of interest.
